# TGF-**β** controls alveolar type 1 epithelial cell plasticity and alveolar matrisome gene transcription in mice

**DOI:** 10.1172/JCI172095

**Published:** 2024-03-15

**Authors:** Danielle A. Callaway, Ian J. Penkala, Su Zhou, Jonathan J. Knowlton, Fabian Cardenas-Diaz, Apoorva Babu, Michael P. Morley, Mariana Lopes, Benjamin A. Garcia, Edward E. Morrisey

**Affiliations:** 1 Division of Neonatology, Department of Pediatrics, Children’s Hospital of Philadelphia, Philadelphia, Pennsylvania, USA.; 2 Penn-CHOP Lung Biology Institute,; 3 Department of Cell and Developmental Biology, and; 4 Department of Medicine, Perelman School of Medicine, University of Pennsylvania, Philadelphia, Pennsylvania, USA.; 5 Penn Cardiovascular Institute, University of Pennsylvania, Philadelphia, Pennsylvania, USA.; 6 Epigenetics Institute, Department of Biochemistry and Biophysics, Perelman School of Medicine, University of Pennsylvania, Philadelphia, Pennsylvania, USA.

**Keywords:** Pulmonology, Extracellular matrix, Integrins, Respiration

## Abstract

Premature birth disrupts normal lung development and places infants at risk for bronchopulmonary dysplasia (BPD), a disease disrupting lung health throughout the life of an individual and that is increasing in incidence. The TGF-β superfamily has been implicated in BPD pathogenesis, however, what cell lineage it impacts remains unclear. We show that *TGFbr2* is critical for alveolar epithelial (AT1) cell fate maintenance and function. Loss of *TGFbr2* in AT1 cells during late lung development leads to AT1-AT2 cell reprogramming and altered pulmonary architecture, which persists into adulthood. Restriction of fetal lung stretch and associated AT1 cell spreading through a model of oligohydramnios enhances AT1-AT2 reprogramming. Transcriptomic and proteomic analyses reveal the necessity of *TGFbr2* expression in AT1 cells for extracellular matrix production. Moreover, TGF-β signaling regulates integrin transcription to alter AT1 cell morphology, which further impacts ECM expression through changes in mechanotransduction. These data reveal the cell intrinsic necessity of TGF-β signaling in maintaining AT1 cell fate and reveal this cell lineage as a major orchestrator of the alveolar matrisome.

## Abstract 2

TGF&beta; mediates alveolar type 1 cell spreading, identity, and matrisome expression during late lung development, which, if perturbed, may predispose to disease including bronchopulmonary dysplasia.

## Graphical abstract

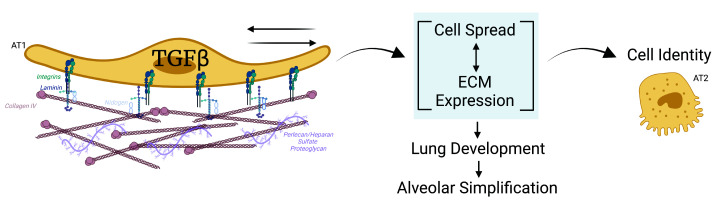

## Introduction

Bronchopulmonary dysplasia (BPD) is a lung disease that disproportionately affects infants born at less than 28 weeks gestation, the threshold for extreme prematurity (1). Despite multiple medical advances including antenatal steroids, surfactant supplements, and improved mechanical ventilation strategies, disease incidence has increased as more infants are born at the limits of viability (2). BPD is characterized as an arrest of alveolarization with enlarged airspaces and thickened septa indicative of a departure from the normal pulmonary developmental programming. This disease not only adversely impacts an infant’s immediate postnatal course during their stay in the neonatal ICU, but it is a life-long disease with increased rehospitalization in the first 2 years of life (3, 4), persistently impaired pulmonary function (5–7), and increased risk of developing chronic obstructive pulmonary disease (COPD) as an adult (8). Although the mechanisms for BPD pathogenesis are not fully known, one candidate that may play a role is transforming growth factor β (TGF-β). Biologically active TGF-β in endotracheal aspirates is abundant in preterm infants and predicts the need for home oxygen therapy (9). Furthermore, elevated levels of TGF-β ligand can be detected in bronchoalveolar lavage fluid from infants with BPD and correlates with disease severity (10). This excess level of TGF-β signaling has been linked to a proinflammatory environment that leads to dysregulated vasculogenesis, which also attracts innate immune cells to further exacerbate the pathology (11). However, the role of TGF-β signaling in the developing lung at homeostasis and how it differentially affects individual cellular niches — particularly the alveolar epithelium — to promote normal development, is unclear.

The TGF-β signaling pathway is responsible for a multitude of critical cellular functions including apoptosis, proliferation, extracellular matrix (ECM) production, and mediating cell fate (12, 13). Importantly, it is also a crucial relay for mechanical signaling within tissues, as inactive TGF-β ligands are embedded within the ECM and become liberated and activated by cellular integrins through mechanical stress (14). TGF-β receptors TGFbr1 and TGFbr2, as well as downstream Smad proteins, exhibit dynamic temporal and spatial expression within the mouse lung, with the highest levels of expression appearing during late lung development (13). Rodent models of TGF-β deletion and overexpression have underscored its importance in regulating lung development (15). Postnatal overexpression of TGF-β1 under an inducible Scgb1a1 promoter or through intranasal delivery of an adenoviral vector resulted in a BPD-like phenotype of alveolar enlargement (16, 17). However, loss of TGFbr2 or downstream components of the signaling pathway including Smad3 is not protective and instead similarly impairs alveologenesis (18–20). Specifically, when TGFbr2 is deleted from undifferentiated alveolar epithelial (AT1) cells early during lung development (E6.5), the lungs demonstrated postnatal alveolar enlargement, whereas deletion in the lung mesenchyme impaired branching morphogenesis (18). Deletion of TGFbr1 during lung endoderm development caused a blockade of secretory cell differentiation in mouse airways (21). Our previous work has shown that TGF-β promotes in vitro cellular spreading of AT1 cells (22). However, what role TGF-β plays in AT1 cell fate decisions or during pulmonary development is unclear.

In the current study, we show that *TGFbr2* deletion in AT1 cells during late lung development increases AT1-AT2 cell reprogramming and induces a BPD-like phenotype of impaired alveolarization and increased septal thickness. A model of oligohydramnios, which predisposes human neonates to pulmonary hypoplasia and increases BPD risk, impairs alveolar stretch and similarly drives AT1-AT2 cell reprogramming. Single-cell RNA-Seq (scRNA-Seq) reveals that expression of numerous genes associated with constituents of the pulmonary ECM and regulatory components, referred to as the pulmonary matrisome, are expressed in AT1 cells starting at the saccular stage through adulthood, reinforcing the AT1 cell as a node for matrisome production that serves as an important hub for intercellular communication. RNA-Seq and proteomics of neonatal AT1 cells reveal that constituents of the pulmonary matrisome are downregulated upon loss of *TGFbr2*. Moreover, TGF-β mediates AT1 cell integrin expression, which in turn affects cell size, morphology, and matrisome gene transcription. These studies reveal that TGF-β signaling is an important regulator of the pulmonary matrisome in AT1 cells, which controls sculpting of the developing alveolus to promote normal lung architecture and function.

## Results

### Loss of TGFbr2 results in increased AT1 cell reprogramming.

**Figure 1:**
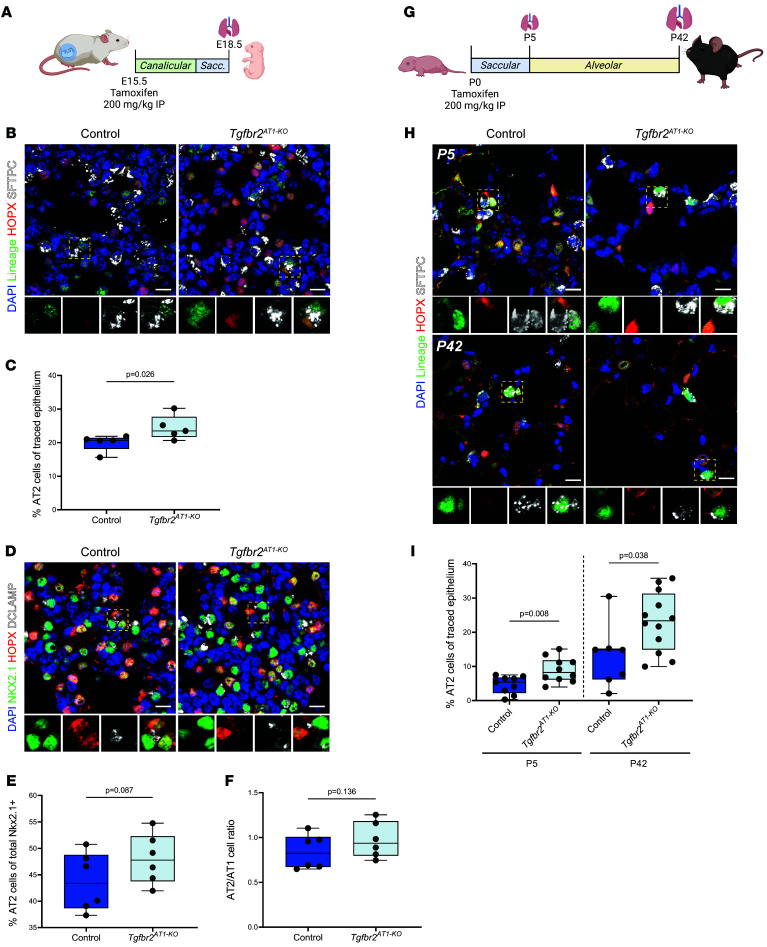
TGF-β is involved in regulating AT1 cell fate during prenatal and postnatal late lung development. (**A**) Tamoxifen was delivered to control heterozygous (*TGFbr2^fl/+^*) littermates and KO (*TGFbr2^fl/fl^ or TGFbr2^AT1–KO^*) mice through IP injection of the pregnant dam at E15.5 and lungs were harvested at E18.5. (**B**) IHC for EYFP, HOPX, and SFTPC demonstrate increased AT1 reprogramming into AT2 cells after prenatal loss of *TGFbr2*. The yellow dashed box denotes the magnified region shown below the image and separated by fluorescence channel. (**C**) Quantification of lineage tracing in **B** denoting percent of cells that were EYFP^+^ and SFTPC^+^ by an unpaired 2-tailed *t* test (*n* = 5 per group). (**D**) IHC for NKX2.1, HOPX, and DCLAMP demonstrate no significant change in the AT1 cell composition after prenatal loss of *TGFbr2*. The yellow dashed box denotes the magnified region shown below the image and separated by fluorescence channel. (**E**) Quantification of AT2 cell numbers from total NKX2.1^+^ cells in (**D**) denoting percent of cells that were NKX2.1^+^ and DCLAMP^+^ and (**F**) the AT2/AT1 cell ratio at E18.5 by an unpaired 2-tailed *t* test (*n* = 6 per group). (**G**) In postnatal lineage-tracing experiments, control (*Hopx^creERT2^:R26R^EYFP^*) and *TGFbr2^AT1–KO^* newborn pups (P0) were injected with tamoxifen and the lungs were harvested at P5 and P42. (**H**) IHC for EYFP, HOPX, and SFTPC demonstrate increased AT1 reprogramming into AT2 cells after prenatal loss of *TGFbr2* at both P5 (top) and P42 (bottom). The yellow dashed box denotes the magnified region shown below the image and separated by fluorescence channel. (**I**) Quantification of lineage tracing in **H** denoting percent of cells that were EYFP^+^ and SFTPC^+^ at P5 (left) and P42 (right) by unpaired 2-tailed *t* tests with Welch’s correction (*n* = 7–12 per group). Each dot represents a single mouse. Scale bars: 10 μm. *P* values are denoted above the plots. Schematics in **A** and **G** were created in BioRender.

To define the function of the main receptor of TGF-β signaling in AT1 cells, TGFbr2 was deleted prenatally (Figure 1, A–F) and postnatally (Figure 1, G–I) using an AT1-specific tamoxifen-inducible transgenic mouse model (*Hopx^creERT2^:TGFbr2:R26R^EYFP^*, hereafter referred to as *TGFbr2^AT1–KO^*). Initially, pregnant dams were injected with tamoxifen at E15.5 followed by fetal lung harvest at E18.5, a point at which the majority of AT1 cells have undergone lineage specification (Figure 1A) (23). These experiments revealed that prenatal loss of *TGFbr2* in AT1 cells yielded an increase in lineage-traced AT2 cells (Figure 1, B and C). There was a trend toward increased AT2 cells as a percentage of total NKX2.1 cells and AT2/AT1 cell ratio (Figure 1, D–F). Postnatal tamoxifen injection at P0 followed by assessment at P5 and P42 also resulted in elevated AT1:AT2 cell reprogramming, albeit to a greater extent at P42, suggesting that the influence of TGF-β on AT1 cell identity may be a permissive rather than direct effect (Figure 1, H and I). Although TGF-β can regulate cellular proliferation, particularly in lung fibroblasts (24, 25) and AT2 cells after injury (26, 27), proliferation of lineage-traced and nonlineage-traced AT2 cells was unaffected by loss of *TGFbr2* (Supplemental Figure 1, A–C; supplemental material available online with this article; https://doi.org/10.1172/JCI172095DS1). The overall numbers of AT1 and AT2 cells at P5 and P42 as a percent of total NKX2.1-positive cells and the AT2/AT1 ratio was also not significantly different (Supplemental Figure 1, E–G). Total NKX2.1^+^ cells per high powered field did not differ at P5 although there was a small increase at P42 in *TGFbr2^AT1–KO^* animals, perhaps driven by a larger proportion of AT1 cells (Supplemental Figure 1H). There was no evidence of increased AT1 cell apoptosis after loss of *TGFbr2* (Supplemental Figure 1I).

To evaluate the effect of *TGFbr2* loss in AT2 cells during development and to better understand how lineage-traced AT2 cells in the *TGFbr2^AT1–KO^* model may differ from nonlineage-traced cells, an AT2-specific tamoxifen-inducible transgenic mouse model (*Sftpc^creERT2^:TGFbr2:R26R^EYFP^*, or *TGFbr2^AT2–KO^*) was employed. As was performed in Figure 1, postnatal tamoxifen injection was performed at P0 followed by analysis at P5 and P42 (Supplemental Figure 2A). There was minimal evidence of AT2-AT1 reprogramming in the neonate, in concurrence with prior work from our lab (Supplemental Figure 2, B and C) (28). Examinations of AT2 cell proliferation in *TGFbr2^AT2–KO^* animals revealed that there was significantly elevated proliferation at P5 that abated by P42 to lower levels than that of the control animals (Supplemental Figure 2, D and E). This increased proliferation was not sufficient to significantly alter the alveolar composition as there was no difference in the numbers of AT2 cells as a percent of total NKX2.1-positive cells or in the AT2/AT1 cell ratio at P5 (Supplemental Figure 2, F–H). However, there was a trend toward decreased AT2 cell numbers of *TGFbr2^AT2–KO^* animals at P42 perhaps secondary to the diminished proliferative capacity seen at this time point.

**Figure 2:**
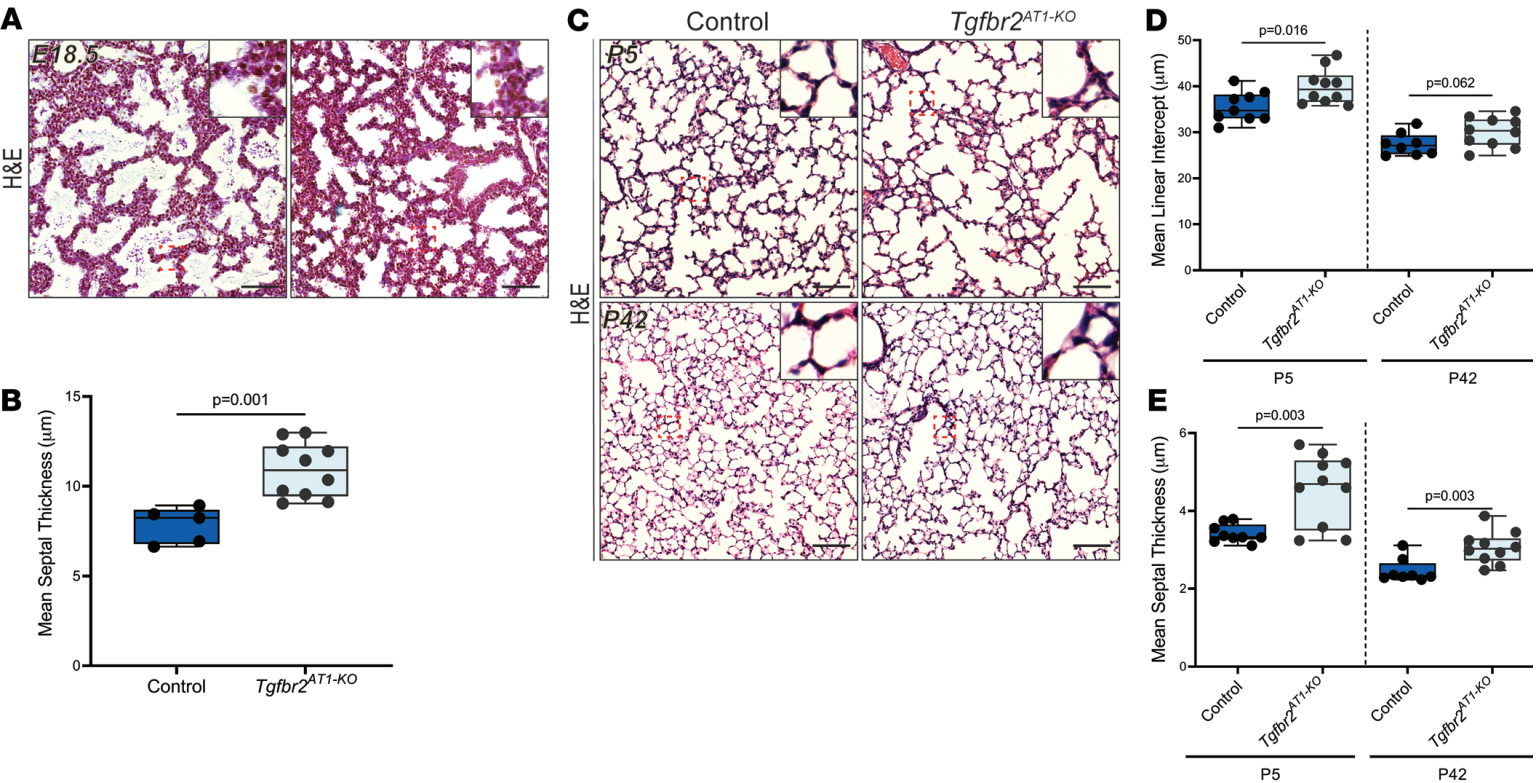
TGF-β is involved in maintaining normal pulmonary architecture during late lung development. (**A**) H&E images of lungs from E18.5 mice following tamoxifen injection at E15.5 show that *TGFbr2^AT1–KO^* embryonic lungs compared with heterozygous littermates demonstrate increased mean septal thickness. Red-dashed boxes indicate the zoomed area shown in the top-right of the image. (**B**) Quantification of mean septal thickness (μm) from **A** by an unpaired 2-tailed *t* test (*n* = 5–10 per group). (**C**) H&E images of lungs from P5 (top) and P42 (bottom) mice following tamoxifen injection at P0 indicate that loss of TGF-β postnatally results in alveolar simplification and increased mean septal thickness. Red-dashed boxes indicate the zoomed area shown in the top-right of the image. (**D**) Quantification of MLI (μm) and (**E**) mean septal thickness (μm) by unpaired 2-tailed *t* tests with Welch’s correction (*n* = 8–10 per group). Each dot represents a single mouse. Scale bars: 50 μm. *P* values are denoted above the plots.

Previous work reported that loss of TGF-β signaling in mouse pulmonary epithelial cells alters pulmonary architecture to induce a BPD-like phenotype (18–20, 29). Prenatal deletion of *TGFbr2* in AT1 cells led to increased septal thickness (Figure 2, A and B). Postnatal loss of *TGFbr2* from birth through P5 and tracked through P42, a time point at which alveologenesis has completed, resulted in alveolar simplification, as demonstrated by increased mean linear intercept (MLI) as well as a more prominent increased mean septal thickness (Figure 2, C–E), indicating that AT1-specific loss of TGF-β during postnatal lung development has lasting effects on pulmonary architecture that persist into adulthood.

### Lack of prenatal mechanical stretch promotes AT1 cellular reprogramming and AT2 cell fate specification.

**Figure 3:**
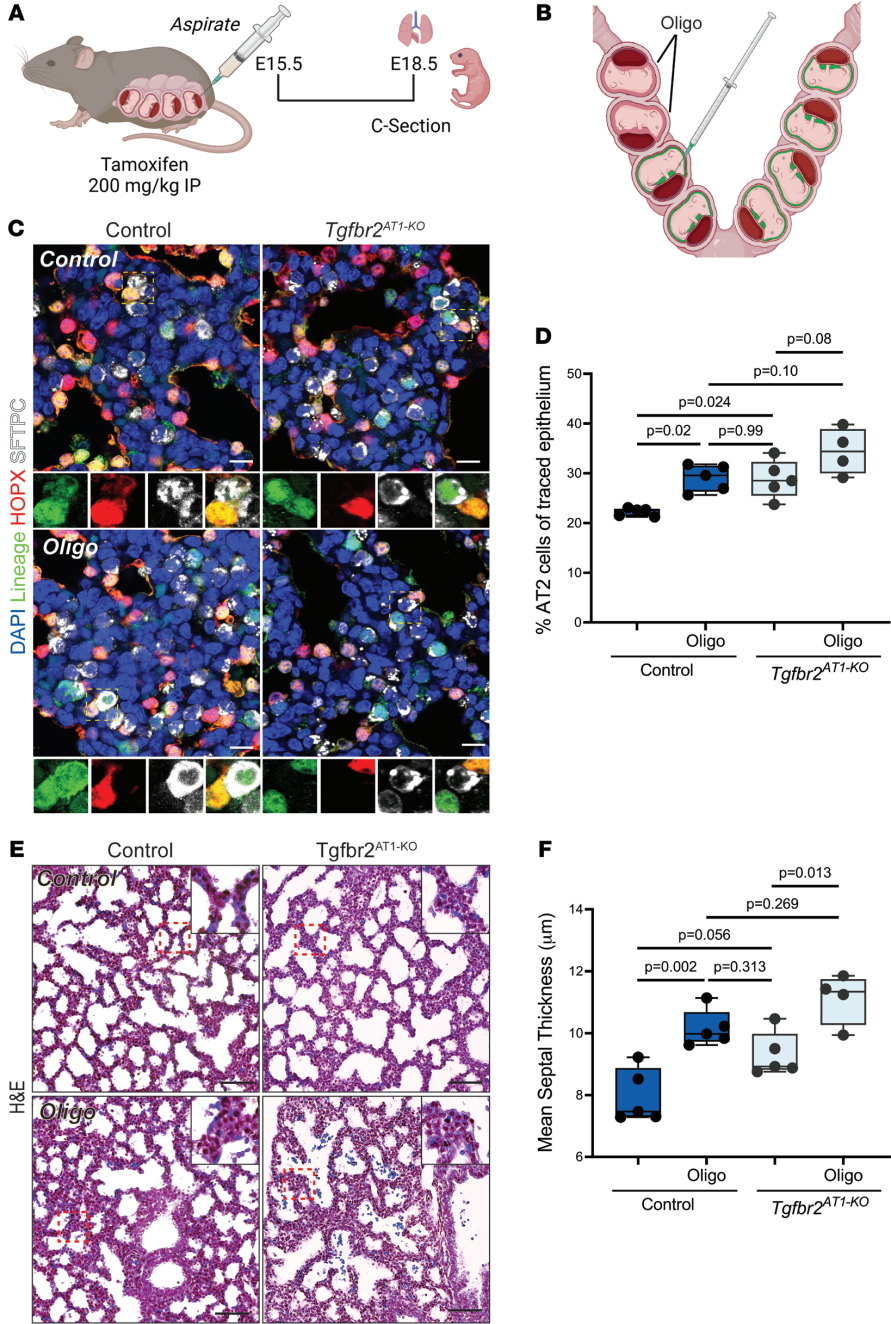
Oligohydramnios alters AT1 cell numbers through increased AT1-AT2 cell reprogramming. (**A**) Oligohydramnios was induced through amniotic fluid reduction at E15.5 followed by injection of tamoxifen to enable lineage tracing. Lungs from the Control or AT1-KO fetal mice were harvested at E18.5 for further analysis. (**B**) Amniotic fluid (green) was aspirated from amniotic sacs in the right uterine horn. (**C**) IHC for lineage-tracing with EYFP, HOPX, and SFTPC demonstrate that there is increased AT1-AT2 cell reprogramming with oligohydramnios although not to a significant extent in AT1-KO pups. The yellow dashed box denotes the magnified region shown below the image and separated by fluorescence channel. (**D**) Quantification of lineage tracing in **C** denoting percent of cells that were EYFP^+^ and SFTPC^+^ by a 2-way ANOVA with Tukey’s post test for multiple comparisons (*n* = 4–5 per group). (**E**) H&E images of control (left) and AT1-KO (right) who underwent oligohydramnios (bottom) or were the littermate controls (top) lungs reveal that lack of amniotic fluid leads to increased mean septal thickness. (**F**) Quantification of mean septal thickness (μm) by a 2-way ANOVA with Tukey’s post test for multiple comparisons (*n* = 4–5 per group). Each dot represents a single mouse. Scale bars: 50 μm for H&E and 10 μm for IHC. *P* values are denoted above the plots. Schematics in **A** and **B** were created in BioRender.

We have shown recently that maintenance of epithelial cell fate is intimately tied to mechanical signals whereby restriction of alveolar stretch in an adult lung deflation model led to reprogramming of AT1 cells into AT2 cells (30). In the human neonate, prenatal reduction of amniotic fluid, termed oligohydramnios, can lead to pulmonary hypoplasia resulting in other respiratory-related morbidities including air leak, pulmonary hypertension, and BPD (31). Although the mechanism for how oligohydramnios directly leads to pulmonary hypoplasia is unclear, it is suspected to involve a restriction of thoracic expansion, thereby altering the intrapulmonary mechanical forces that are essential to distend the airways and promote normal lung maturation (32, 33). A previous murine oligohydramnios model in which amniotic fluid was reduced at E15.5 followed by analysis at E18.5 resulted in reduced transcription of the AT1 cell-associated gene *Pdpn* and elevated expression of the AT2 cell marker *Sftpc* compared with nontreated littermates (34). We performed a similar series of experiments with the inclusion of a lineage trace for AT1 cells (Figure 3, A and B) and the *TGFbr2^AT1–KO^* mouse model. Oligohydramnios resulted in increased AT1-AT2 cell reprogramming (Figure 3, C and D). Of note, AT1-AT2 reprogramming from oligohydramnios was not significantly elevated between *TGFbr2^AT1–KO^*-oligo pups and their control littermates (Figure 3D). In addition, oligohydramnios resulted in increased mean septal thickness (Figure 3, E and F) as well as an increase in the percentage of AT2 cells per NKX2.1^+^ epithelium and increased AT2/AT1 cell ratio in both genotypes (Supplemental Figure 3). However, loss of TGFbr2 in AT1 cells did not affect the AT1 cell composition (Supplemental Figure 3). Combined, these data indicate that inhibiting essential mechanical forces during late pulmonary development is sufficient to alter epithelial cell fate in the fetal lung.

### AT1 cells are enriched in genes associated with the pulmonary matrisome and are controlled by TGF-β.

**Figure 4:**
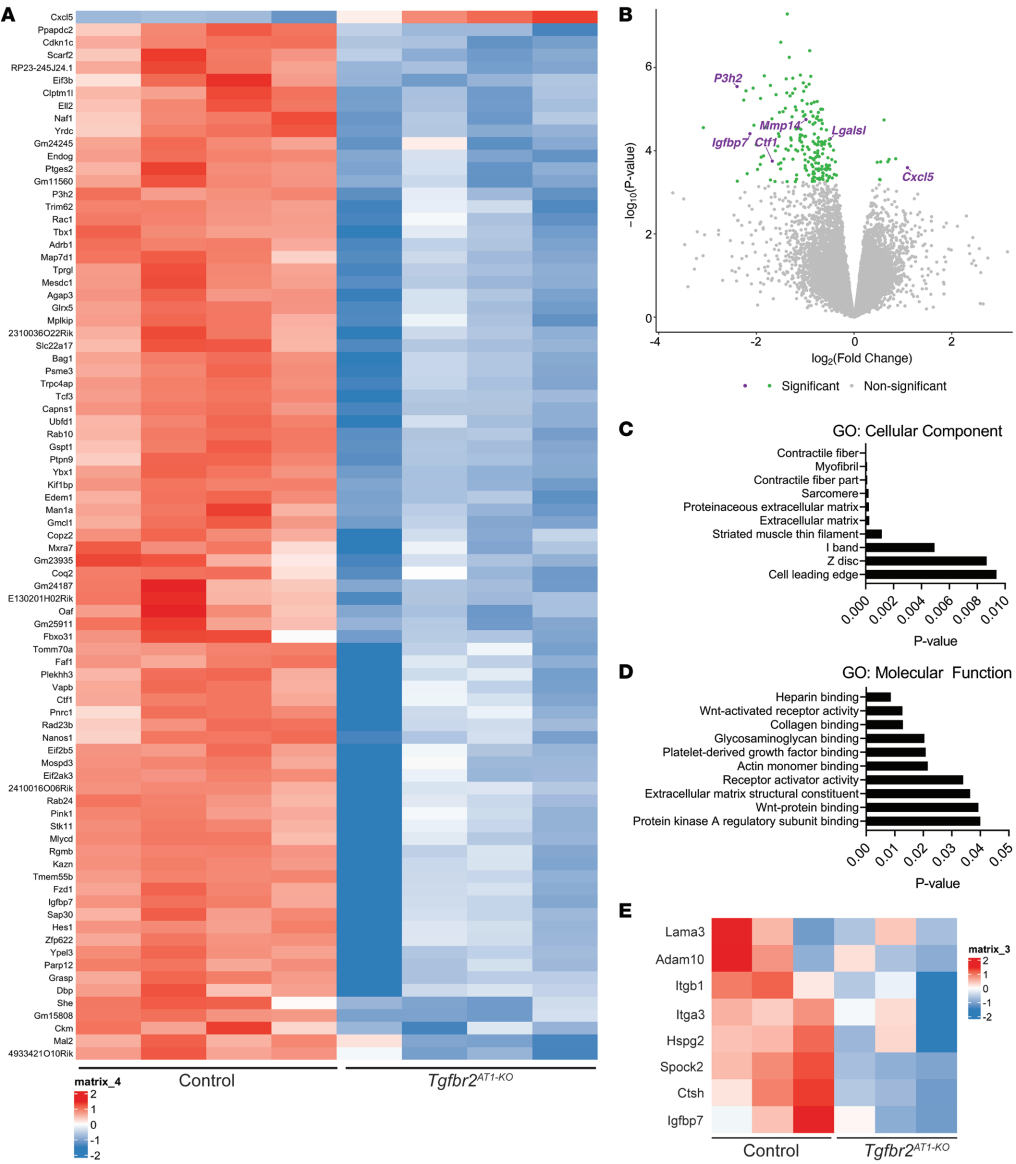
Transcriptomic and proteomic profiling reveal a role for TGFβ in regulating AT1 cell expression of the pulmonary matrisome. (**A**) Heatmap of differentially expressed genes (upregulated on top, downregulated on bottom) from AT1 cells of control heterozygous littermates (left) or *TGFbr2^AT1–KO^* (right) mice at P5 (*n* = 4). Genes depicted were filtered with a cut off of 0.05 for the *P* value and log FC of 2. (**B**) Volcano plot of differentially expressed genes from RNA-Seq from (**A**) with ECM-related genes labeled reveal that several are significantly downregulated with loss of *TGFbr2*. (**C**) GO enrichment for cellular component and (**D**) molecular function of downregulated genes reveal several ECM-related terms. (**E**) Heatmap of ECM-related proteins from proteomics results of control heterozygous littermates (left) or *TGFbr2^AT1–KO^* (right) AT1 cells obtained at P5 (*n* = 3).

To determine gene expression changes due to loss of *TGFbr2* in AT1 cells, RNA-Seq of sorted AT1 cells from neonatal *TGFbr2^AT1–KO^* mice and heterozygous littermates at P5 was performed. One of the most significantly downregulated genes associated with *TGFbr2* loss was *P3h2*, a member of the prolyl 3-hydroxylase subfamily that controls posttranslational 3-hydroxylation of proline residues on collagen IV, which is necessary for collagen cross-linking and stability (35). Because TGF-β is a known modulator of both ECM production and maintenance in the lung (15, 36–38), we interrogated the data set for other matrisome-related genes (39). We found that, in addition to *P3h2*, an additional ECM regulator, *MMP14*, was downregulated, as well as the ECM glycoprotein *Igfbp7* and matrisome-associated secreted factors *Lgals1* and *Ctf1*. *Cxcl5*, a secreted factor involved in neutrophil recruitment after injury, was upregulated (Figure 4, A and B). Evaluation of gene ontology pathway enrichment revealed that loss of *TGFbr2* in AT1 cells during late lung development was associated with enrichment of cellular components of the ECM and genes involved in ECM binding (Figure 4, C and D). We next assessed changes in the proteome by mass spectrometry analysis. These studies revealed downregulated expression of several ECM-related proteins including glycoproteins Lama3 and Igfbp7, proteoglycans Spock2 and Hspg2, ECM regulators Adam10 and Ctsh, and integrins Itga3 and Itgb1 (Figure 4E).

**Figure 5:**
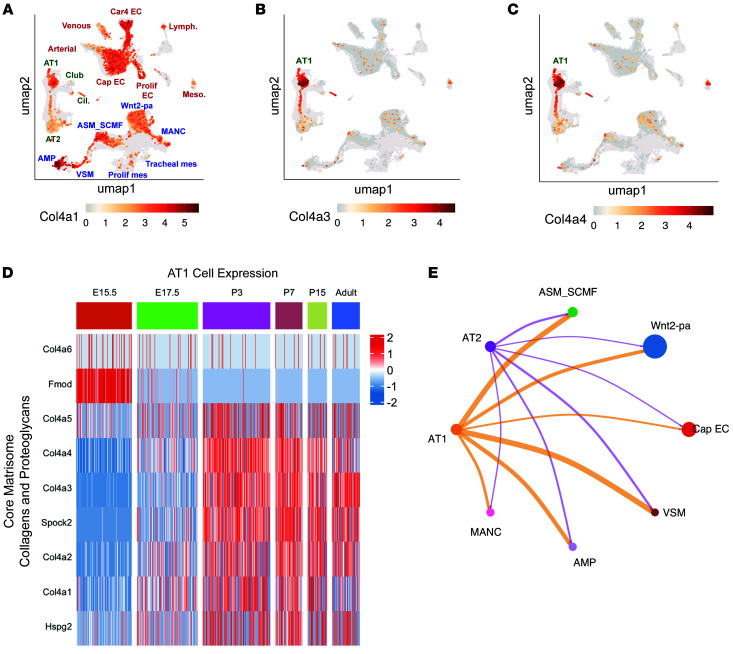
AT1 cells express collagen 4 in a time-dependent manner and are important hubs for communication across the developing lung. (**A**) UMAP of scRNA-Seq data at P3 from Zepp et al, 2021 (40) of collagen IV subtype expression across the mouse lung including *Col4a1*, (**B**) *Col4a3*, and (**C**) *Col4a4*, denoting that the latter are highly specific for AT1 cells. (**D**) Evaluation of previously generated scRNA-Seq data across development indicates that AT1-enriched collagen IV and proteoglycan genes become highly expressed in the postnatal period through adulthood indicating a role for AT1 cells in the active production and remodeling of the pulmonary matrisome across the lifespan. (**E**) Circle plot from CellChat analysis of outgoing communication from AT1 and AT2 cells at P3 (from Zepp et al, 2021 data set) (40), to cells of the mesenchyme (ASM_SCMF, Wnt2-pa, VSM, AMP, and MANCs) and to the endothelium (CapEC), denoting that AT1 cells are the primary communicators of the alveolar epithelium.

Transcriptomic and epigenetic profiling of AT1 cells has revealed that they are highly enriched for pathways involving focal adhesion, integrin-mediated cell adhesion, and cytoskeleton regulation (28, 40). scRNA-Seq of the developing lung has highlighted the importance of AT1 cells in transcription of ECM-related genes including collagens, proteoglycans, and glycoproteins (40, 41). *Col4a3* and *Col4a4* as well as Laminin-332 constituents *Lama3*, *Lamb3*, and *Lamc2* have higher expression in AT1 cells compared with other cell populations within the lung (41). Indeed, when comparing Col4 subtypes at P3, *Col4a3*, and *Col4a4* are highly specific to AT1 cells when compared with *Col4a1*, which is also present at high levels in the endothelium and mesenchyme (Figure 5, A–C). These data have been corroborated by other studies (41). Using a previously generated scRNA-Seq data set, we compared other AT1-enriched core matrisome constituents across development including collagens and proteoglycans (Figure 5D) as well as glycoproteins (Supplemental Figure 4A) (39, 40). Most AT1 cell–enriched matrisome-related genes become highly expressed at E17.5, corresponding with the start of the saccular stage of lung development. Nearly all of these genes remain highly expressed through adulthood, indicating a life-long importance of AT1 cells as critical orchestrators for the production and maintenance of the pulmonary matrisome. Similarly, integrin expression in AT1 cells also increases starting at E17.5 and remains elevated through adulthood with the exception of the laminin-binding integrin Itga6, which appears to exhibit less expression postnatally (Supplemental Figure 4B).

Using this same scRNA-Seq data set, we performed receptor-ligand analysis with CellChat to identify incoming and outgoing signals across the developing lung at P3. Figure 5E shows a circle plot of outgoing signals from AT1 cells to subclusters of the endothelium and mesenchyme. AT1 cells demonstrate a more robust outgoing signaling network compared with AT2 cells, highlighting them as the primary drivers of communication within the alveolar epithelium. Interrogating those outgoing signals to identify receptor partners reveals that a significant portion of the predicted intercellular communication from AT1 cells to the mesenchyme arises from the basement membrane constituents, particularly the AT1 cell–enriched subtypes Col4a3 and Col4a4 (Supplemental Figure 5).

**Figure 6:**
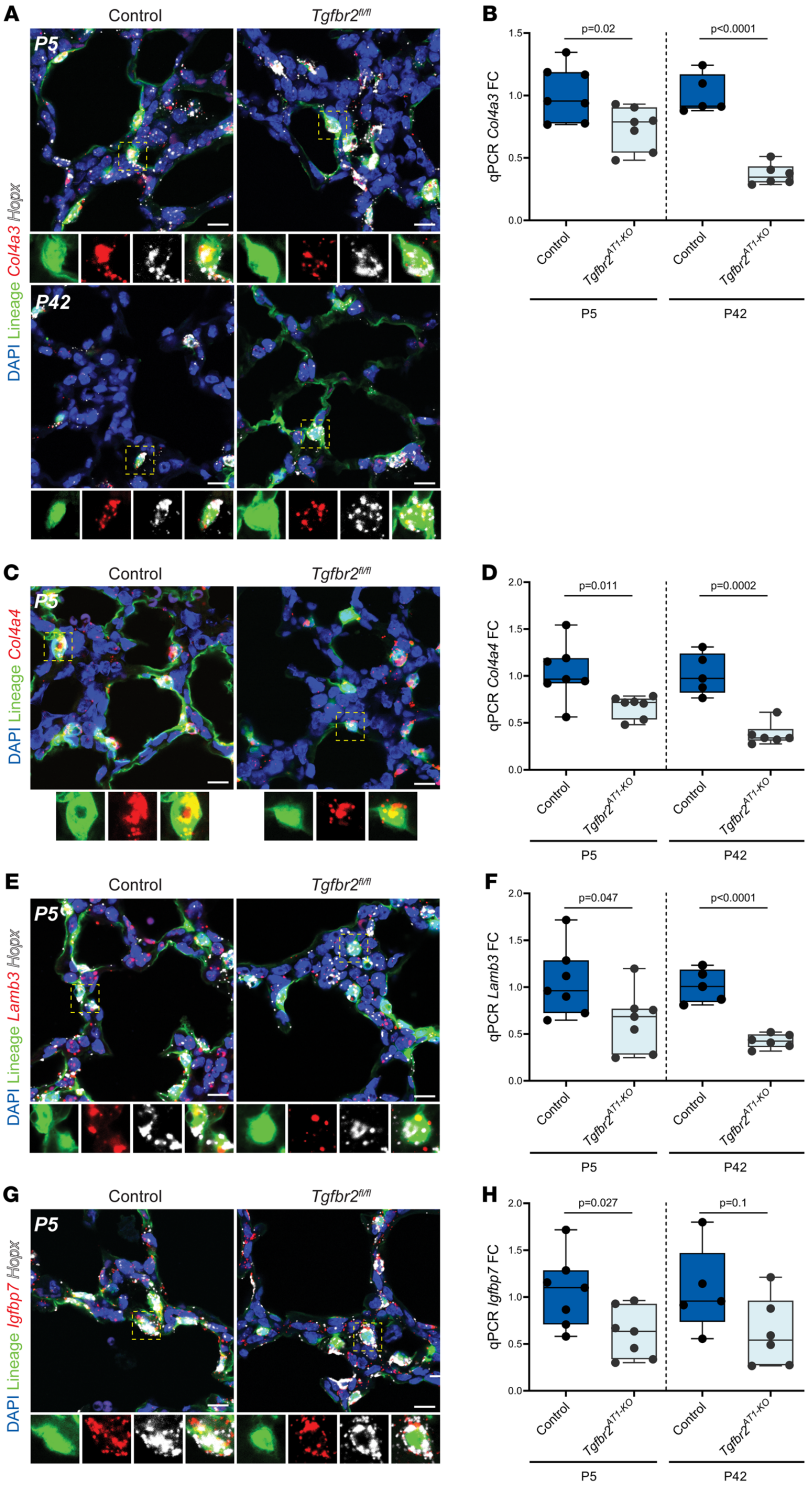
Loss of TGF-β at birth perturbs AT1-mediated matrisome expression through adulthood. (**A**) RNA FISH for *Col4a3* and *Hopx* with IHC for lineage-labeled cells at P5 (top) and P42 (bottom). The yellow dashed box denotes the magnified region shown below the image and separated by fluorescence channel. (**B**) *TGFbr2^AT1–KO^* AT1 cells exhibit decreased qPCR RNA (FC compared with GAPDH, normalized to controls) transcript expression for several AT1 cell-enriched core matrisome constituents including collagens *Col4a3* and (**D**) *Col4a4* and glycoproteins (**F**) *Lamb3* and (**H**) *Igfbp7 at* P5 that persists to P42 (*n* = 5–7, unpaired 2-tailed *t* test with Welch’s correction). (**C**) RNA FISH of P5 lungs for *Col4a4* with IHC for lineage-labeled cells. The yellow dashed box denotes the magnified region shown below the image and separated by fluorescence channel. (**E**) RNA FISH for *Hopx* and glycoproteins *Lamb3* and (**G**) *Igfbp7* with IHC for lineage-labeled cells. The yellow dashed box denotes the magnified region shown below the image and separated by fluorescence channel. Each dot represents a single mouse. Scale bars: 10 μm. *P* values are denoted above the plots.

Because of the importance of core matrisome components including collagens, proteoglycans, and glycoproteins as well as ECM regulators to AT1 cell biology, we elected to expand our scope beyond what was seen in the informatics analysis to evaluate additional matrisome components in *TGFbr2*-deficient AT1 cells. The specific collagen and laminin subtypes were chosen due to their high expression in AT1 cells as depicted in scRNA-Seq data (40, 41). The Col4 and laminin-332 genes were decreased on qRT-PCR analysis in TGFbr2^AT1–KO^ cells, although to a greater extent at P42 (Figure 6, A–F and Supplemental Figure 3A). Corresponding *Col4a3* and *Col4a4* RNA-scope indicated the relative specificity for each transcript in the *Hopx*^+^-labeled cells compared with *Lamb3*, which is present in several other nonlabeled cells in agreement with data from scRNA-Seq (Figures 6, A, C, and E). The remaining genes were chosen due to their presence in the RNA-Seq and/or proteomics data and included the glycoprotein *Igfbp7*, proteoglycan *Hspg2*, and ECM regulator *P3H2*. *Plod2*, a gene that is involved in collagen hydroxylation and crosslinking, was chosen, as it is known to be regulated by TGF-β (42). Importantly, expression of each of these genes as determined by qRT-PCR was decreased in the TGFbr2-deficient AT1 cells from neonates and, most significantly, in those same cells in adults (Figure 6, G and H and Supplemental Figure 6, B–D).

### TGF-β affects AT1 cell spreading and morphology through transcriptional regulation of integrins.

**Figure 7:**
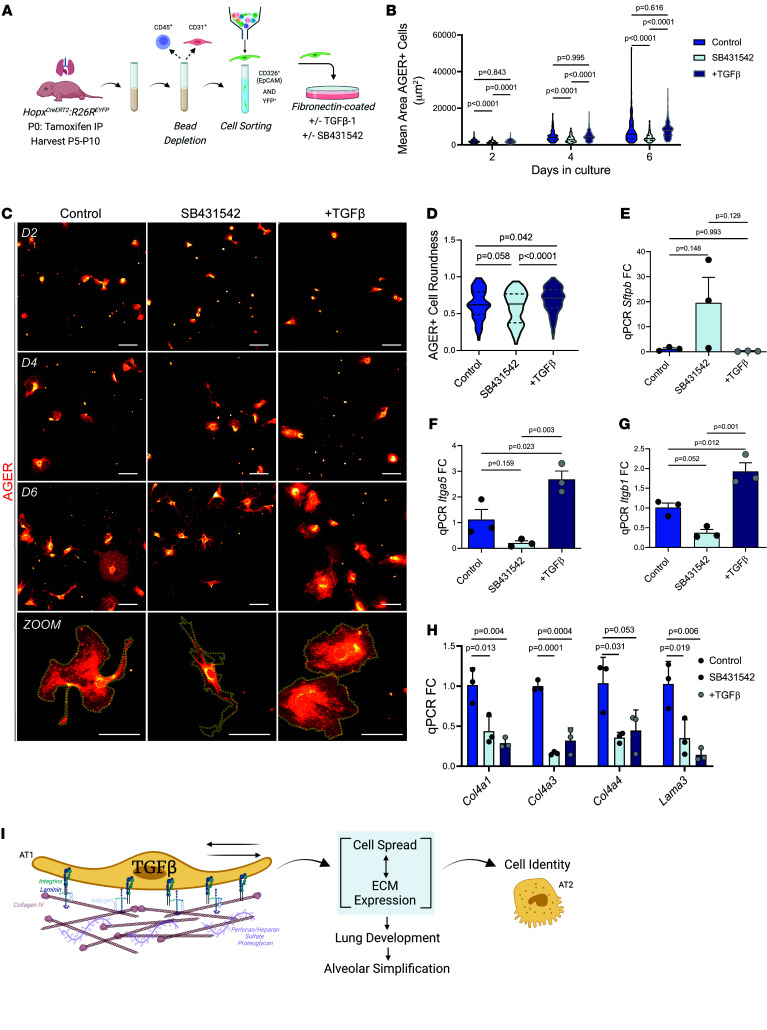
TGF-β–mediated integrin binding regulates AT1 cell size, morphology, and ECM expression. (**A**) To obtain AT1 cells for culture, P5–P10 pup lungs were obtained after lineage labeling with tamoxifen at P0. Whole lung cell suspensions were obtained using a dispase, DNase, and collagenase digestion buffer after which the epithelial cell population was enriched by depleting the CD45^+^ and CD31^+^ population. Remaining cells were fluorescently sorted with FACS to obtain a CD326^+^ and YFP^+^ suspension. Cells were plated onto fibronectin-coated plates with or without TGF-β1 ligand (7.5 ng/mL) or TGF-β inhibitor SB431542 (10μM) and evaluated at days 2, 4, and 6. (**B**) ICC of RAGE^+^ (AGER^+^) cells treated with TGF-β ligand or inhibitor in culture at days 2, 4, and 6 with a zoomed image of cells at day 6 appearing at the bottom. Scale bars: 100 μm. (**C**) Quantification of mean AGER^+^ cell area depicted in **B** by 1-way ANOVA with Holms Šidák’s test for multiple comparisons (*n* = 135–231). (**D**) Quantification of mean cell roundness at day 6 depicted in **B** by 1-way ANOVA with Holms Šidák’s test for multiple comparisons (*n* = 148–167). (**E**) Quantification of qPCR RNA transcript expression levels (FC compared with GAPDH, normalized to controls) of the AT2 marker *Sftpb*, fibronectin-binding integrins (**F**) *Itga5* and (**G**) *Itgb1*, and (**H**) basement membrane constituents including the collagen IV subtypes *Col4a1*, *Col4a3*, and *Col4a4* and laminin-332 constituents *Lama3* and *Lamb3* (*n* = 3 per group, 1-way ANOVA with Tukey’s multiple comparisons). (**I**) Representative schematic of findings indicating that TGF-β regulates integrin expression to guide ECM binding and cellular spread, which affects cell identity and matrisome expression and impacts lung development. Schematics in **A** and **I** were created in BioRender. Results are representative of 3 experiments.

Prior work from our lab has established a role for TGF-β in promoting AT1 cell spreading (43). We wanted to evaluate the consequence of increased or inhibited TGF-β signaling on AT1 cell spreading as well as their effects on matrisome transcription. We isolated AT1 cells from neonatal mice and generated primary AT1 cell cultures, which were treated with TGF-β1 ligand or the TGF-β inhibitor SB431542 (Figure 7A). Cell size was quantified every other day of culture up to 6 days and RNA was collected for analysis. The cultured cells expressed the AT1 cell marker Ager and gradually spread through the course of the experiment (Figure 7, B and C). Treatment with SB431542 decreased the extent of AT1 cell spreading while exogenous TGF-β1 ligand had no significant affect (Figure 7C). In contrast, there was a difference in cell morphology upon treatment with exogenous TGF-β1 (Figure 7, B and D). While the cells treated with SB431542 spread less but acquired a more stellate appearance, the cells treated with exogenous TGF-β1 appeared rounder and demonstrated more uniform spreading compared with the controls (Figure 7, B and D). This alteration in cell shape was evaluated by measuring cell roundness with the Fiji imaging software package, which confirmed that TGF-β1–treated cells had a higher roundness score compared with the control and inhibitor-treated cells (Figure 7, B and D). Expression of the AT2 cell marker *Sftpb* was higher in cells treated with inhibitor although not statistically significant (Figure 7E). Cells from AT1-KO animals also demonstrated a significantly reduced spreading capacity compared with control cells, even when treated with TGF-β1 ligand (Supplemental Figure 7, A and B). Further, plating of AT1 cells on Col4 substrate yielded a similar pattern of cellular spreading upon addition of inhibitor and TGF-β1 ligand (Supplemental Figure 7C). Moreover, there was a significantly increased spreading capacity on Col4 with TGF-β1 ligand compared with untreated cells (Supplemental Figure 7D).

We examined whether expression of integrins and AT1 matrisome components identified in our in vivo loss-of-function studies were altered in cultured AT1 cells treated with TGF-β1 ligand or inhibitor. Treatment with TGF-β1 significantly increased both *Itga5* and *Itgb1* expression compared with the control and inhibitor-treated groups, while inhibition decreased their expression (Figures 7, F and G). *Itgb1* expression similarly increased in cells plated on Col4 (Supplemental Figure 7E). Loss of TGF-β signaling in cultured AT1 cells led to decreased expression of *Col4a1*, *Col4a3*, *Col4a4*, *Lama3*, and *Lamb3* (Figure 7H). Surprisingly, addition of exogenous TGF-β1 led to a similar decrease in expression of these matrisome components (Figure 7H). These data suggest that AT1 cells respond to TGF-β activation via a negative feedback loop, which suppresses expression of some matrisome components, likely to dampen excessive ECM deposition. Together, these data support the important function of TGF-β as a mediator of AT1 cellular spreading through integrin-binding to the ECM. AT1 spreading and its associated changes in mechanotransduction are intimately tied to AT1 cell identity and the ability of these cells to function as a node for matrisome expression in the lung (Figure 7I).

## Discussion

In this study, we demonstrated the importance of TGF-β signaling in AT1 cell fate, which, when disrupted, leads to a BPD-like phenotype with enlarged alveoli and increased septal thickness. Using a model of mouse oligohydramnios, which limits lung prenatal cyclical stretch, we showed that the fate of prenatal AT1 cells is exquisitely sensitive to the natural breathing movements exhibited by the mammalian lung prior to birth. TGF-β signaling mediated the expression of multiple members of the pulmonary matrisome in AT1 cells including integrins, which are necessary to guide and sculpt the emerging alveolus during late lung development. Ex vivo modeling of AT1 cell spreading showed that TGF-β signaling impacted both AT1 cell morphology and overall extent of spreading, through integrin-mediated binding to the ECM, which was sensitive to negative feedback from excessive TGF-β activity. These data highlight AT1 cells as a nodal organizing center for sculpting and remodeling the lung alveolus during late development through TGF-β–mediated expression of the pulmonary matrisome.

RNA-Seq shows that AT1 cells are enriched for pathways involving focal adhesion, the actin cytoskeleton, ECM-receptor interactions, Hippo signaling, and TGF-β signaling, which are all related to mechanotransduction (28, 30). Neonatal loss of YAP/TAZ in AT1 cells results in a striking increase in AT1-AT2 cellular reprogramming and alveolar simplification (28). Further, in vivo loss of interactions between the cytoskeleton and ECM through Cdc42 and Ptk2 in adult AT1 cells promotes AT1-AT2 cell reprogramming (30). Conversely, physically constraining AT2 cells from spreading in vitro inhibits their AT2-AT1 differentiation (30). Blockade of stretch during breathing through bronchial ligation also results in AT1-AT2 cell reprogramming, underscoring the essential role of mechanical signaling to maintain AT1 cell fate (30). Impaired integrin expression in the present *TGFbr2* loss-of-function study alters AT1 cell spreading ability, which affects expression of basement membrane constituents as well as enzymes for posttranslational processing to ensure normal morphology. These changes further impair normal AT1 cell spreading, which promotes cellular reprogramming and a greater exacerbation of altered matrisome maintenance, which can lead to pathologic alveolarization and defects in secondary septation. Likely, alterations in other important components of cell-ECM interactions, namely Sdc4 and Sdc1, which were identified in the CellChat analysis (Supplemental Figure 5) and interact with a wide array of ligands and the cytoskeleton, will also have implications on AT1 cell spreading capacity, identity, and function (44). These data support the emerging concept that AT1 cells are an organizing node for promoting and maintaining lung alveolar architecture.

In other organ systems including bone, which is under constant mechanical load, mechanotransduction is intimately tied to ECM expression in order to provide the necessary structure and support (45). The lung, which is also exposed to continuous mechanical stress through cyclical breathing, likely regulates ECM production through constant sensing of mechanical strain in the various tissue niches. While several studies have explored ECM remodeling within the lung and the role of TGF-β, these have focused predominantly on the profibrotic response of mesenchymal cells and AT2 cells in disease states such as cancer and idiopathic pulmonary fibrosis (IPF) (20, 33, 46, 47). The novel role for AT1 cells as critical hubs for expression of core matrisome constituents, regulatory enzymes, and secreted factors during development has recently been described (40, 41). AT1 cells produce constituents of the basement membrane, including collagen IV, laminins, and other glycoproteins and proteoglycans that are important to maintain the structure and function of the alveolar-capillary barrier. Some components, including *Col4a3, Col4a4,* and *Col4a5*, which make up the collagen IV α3α4α5 triple helix, and laminin-332 exhibit the greatest expression in AT1 cells compared with other cell types within the lung (41). The collagen IV α3α4α5 isoform is thought to be stiffer owing to an increased number of crosslinks (48). Furthermore, the collagen IV α3α4α5 isoform in the adult renal glomerular basement membrane (GBM) has been explored due to its role in Alport disease, in which mutations in this isoform lead to progressive renal failure (49). Goodpasture’s syndrome, another progressive renal disease in which antibodies against Col4a3 attack the basement membrane, results in pulmonary manifestations including hemoptysis and pulmonary hemorrhage (50). Therefore, production and maintenance of the alveolar basement membrane by AT1 cells likely serves a critically important role to maintain the integrity of the alveolar-capillary barrier and promote cell spreading during alveologenesis to increase surface area for gas exchange. Beyond matrisome constituents, AT1 cells also influence neighboring cells through paracrine signaling of matrisome-related secreted factors that likely further guide pulmonary development and remodeling. Given the focal enrichment of AT1 matrisome expression in the adult, it is likely that the AT1 cell plays a key role in maintaining ECM-driven alveolar architecture maintenance and also may play an important role in fibrotic diseases of the lung such as IPF.

The lung is an organ under constant physical strain during normal breathing motions and displays remarkable regenerative capacity when challenged by injury. Late lung development marks a time of vulnerability in which the lung may be required to deviate from normal developmental programming and simultaneously respond to additional insults. Although the majority of injury models in neonates and adults involve infection, fibrosis, or hyperoxia, less is known regarding the effects of altered biophysical force, particularly during prenatal lung development. However, the potential clinical implications are important. There are numerous congenital anomalies that induce pulmonary hypoplasia including congenital diaphragmatic hernia, giant omphalocele, musculoskeletal deformities, genitourinary blockage, and renal dysplasias. Critically, loss of amniotic fluid from preterm premature rupture of membranes and subsequent premature birth can lead to an increased risk for BPD, increased hospitalization during the first few years of life, and perinatal death in extremely preterm infants (51–53). Conversely, elevated biophysical force through exposure to mechanical ventilation induces TGF-β signaling, leading to proinflammatory activity and disordered collagen, which can influence BPD pathogenesis in preterm infants. Future studies on the impact of mechanical stress on AT1-mediated matrisome maintenance will likely identify additional pathways that drive aberrant remodeling and intercellular communication during development and predisposes patients to diseases such as BPD and pulmonary hypoplasia.

## Methods

### Animals.

*Hopx^CreERT2^* [Jackson Labs no. 017606] (54), *R26R^EYFP^* (Jackson Labs no. 007903) (55), and *Tgbr2^fl/fl^* (Jackson Labs no. 012603) (56) have been previously described. Sftpc^creERT2^ mice were a gift from the Chapman lab (University of California, San Francisco, San Francisco, California, USA) (57). All mice were maintained on a mixed background (C57BL/6J and CD-1) and all experiments were of mixed sex. Controls were either heterozygous littermates or mice without the floxed allele, as indicated in the text and figure legends.

### Lineage tracing.

Neonatal lineage tracing experiments were performed as previously described (23, 28, 40, 58). In studies of late lung development, pregnant dams at E15.5 gestation or newborn pups at P0 were injected intraperitoneally with a single 200 mg/kg dose of a solution containing tamoxifen, 10% ethanol, and 90% corn oil (all from Sigma-Aldrich).

### Oligohydramnios model.

Amniotic fluid removal of pregnant dams at E15.5 to induce oligohydramnios and pulmonary hypoplasia of the embryos was performed as described in previous publications (34, 59). Pregnant mice were anesthetized with isoflurane and prepared in sterile fashion. A longitudinal incision was made along the abdomen revealing the embryos, and amniotic fluid was removed from embryos of the right uterine horn while those of the left remained untouched to serve as littermate controls. The mother was sutured closed and 200 mg/kg of tamoxifen was delivered intraperitoneally for lineage tracing experiments. At E18.5 the mother was euthanized with CO_2_ and the lungs of the progeny were removed for analysis.

### AT1 cell isolation from lungs.

Lungs from tamoxifen-treated animals were removed and digested in a solution containing collagenase (Thermo Fisher Scientific), DNase (Thermo Fisher Scientific), and dispase (Corning), as previously described (28, 40). Red blood cells were lysed using ACK buffer and cells were then stained with EpCAM-PE-Cy7 (eBioscience, G8.8, 1:200), CD31-APC (eBioscience, 390, 1:200), and CD45-APC (eBioscience, 30-F11, 1:200). The CD45^+^ and CD31^+^ stained-cells were depleted on MACS LS columns after incubation with anti-APC magnetic beads (Miltenyi biotech) leaving an enriched epithelial cell population. The cell-containing solution was then sorted with a BD FACS Jazz cell sorter (Becton Dickinson) for YFP^+^ and CD326^+^ (EpCAM) double-positive cells to obtain lineage-traced AT1 cells for further analysis including RNA-Seq, proteomics, qRT-PCR, and in vitro studies.

### Histology and alveolar measurements.

Animals were euthanized with CO_2_ and a thoracotomy was done to allow access to the lungs. The heart was perfused with cold PBS followed by inflation of the lungs with 2% paraformaldehyde at 25 cm H_2_O. The lungs were fixed overnight in 2% paraformaldehyde after which they were embedded in paraffin and sectioned at 6μm thickness. For alveolar morphology analysis, slides were stained with H&E per standard protocol. Images were obtained with a Nikon Eclipse 80i microscope or EVOS FL Auto2 Imaging System. MLI was performed with Fiji software across 10 images at 20 × magnification per sample, counting the number of intercepts across 400 μm on 6 horizontal lines. Mean septal thickness was performed by measuring septal thickness in Fiji across 20 septa per image and 10 images per sample to yield 200 individual measurements that were averaged.

### IHC and RNA fluorescence in situ hybridization.

IHC was performed with the following antibodies: Cleaved Caspase 3 (rabbit, R&D Systems, MAB835, 1:100), HOPX (mouse, Santa Cruz, sc-398703 1:00), GFP (chicken, Aves Labs, GFP-1020, 1:200 or goat, Abcam, ab6673, 1:200), Ki67 (mouse, BD Biosciences, 550609, 1:200), Lamp3 (DC-Lamp) (rat, Novus, DDX0191P-100, 1:100), NKX2.1 (Ttf1) (rabbit, Abcam, ab76013, 1:50), and SFTPC, (rabbit, Millipore Sigma, AB3786, 1:100). Slides were mounted using Slowfade Diamond Antifade Mountant (Invitrogen, catalog # S36972). RNA fluorescence in situ hybridization (FISH) was performed with RNAscope according the manufacturer’s instructions with the following probes: Mm-Col4a4-C2 (no. 1078041-C2), mm-Col4a3 (no. 544361), mm-Hopx-C2 (no. 405161-C2), mm-Igfbp7 (no. 425741), Mm-Lamb3 (no. 552161), and mm-Sftpc-C2 (no. 314101-C2). Fluorescent imaging was performed on a Zeiss LSM 710 confocal microscope with a 40 × water-immersion objective and processed using Fiji software (60). For cellular quantification of z-stacked images, at least 5 images or 200 cells in total were assessed per sample.

### RNA-Seq.

AT1 cells were isolated from neonatal mouse lungs at P5 as described. RNA isolation was done with the PureLink RNA Micro kit (Thermo Fisher Scientific) and library preparation was performed with the NEBNext Single Cell/Low Input RNA Library Prep Kit for Illumina (New England Biolabs, catalog no. E6420) following the manufacturer’s instructions. Libraries were sequenced using the Illumina HiSeq. Analysis was performed as previously described (61, 62). In brief, the FastQC program was used to assess quality of Fastq files before aligning to the mouse reference genome (GRCm39) using the STAR aligner (63). The MarkDuplicates program from Picard tools was used to flag duplicate reads and duplicates were excluded before computing per gene read counts for Ensembl (v104) gene annotations, using the Rsubread R package. Gene counts were normalized with the TMM method in the edge R package, and genes with 25% of samples with a counts per million (CPM) under 1 were deemed low expressed and removed (64). Expression levels were transformed using VOOM in the limma R package. The limma R package was used to generate a linear model to perform differential gene expression analysis using linear models (65). Given the small sample size of the experiment, we used the empirical Bayes procedure as implemented in limma to adjust the linear model fit and to calculate *P* values. These *P* values were adjusted for multiple comparisons using the Benjamini-Hochberg procedure. Plots were generated in R and GO enrichment was performed using the GAGE R package. The time course of scRNA-Seq genes in AT1 cells was derived from previously generated data (40) and heatmaps were generated in R. Receptor-ligand analysis was generated with CellChat and plots were generated in R (66). Matrisome-specific genes were derived from the Matrisome Project (39).

### Proteomics.

Cells were lysed for 5 minutes on ice in 20 μL of lysis buffer containing 8 M urea, 0.1 M sodium chloride, and 50 mM Tris, pH 8.0, supplemented with phosphatase and protease inhibitors. Samples were centrifuged at 17,900*g* for 5 minutes and the supernatant containing proteins was transferred to a new tube. Proteins were reduced using 5 mM DTT for 1 hour at room temperature and alkylated with 20 mM iodoacetamide (IAA) in the dark for 30 minutes at room temperature. After that, samples were diluted with 4 volumes of 0.1 M ammonium bicarbonate and digested with 0.5 μg of trypsin overnight at room temperature. Samples were desalted and resuspended in 10 μL 0.1% formic acid for analysis (5 μL injections) by nLC-MS/MS, composed of an EASY-nLC 1,000 (Thermo Fisher Scientific) coupled to a Q Exactive Orbitrap mass spectrometer (Thermo Fisher Scientific) operating in data-dependent–acquisition (DDA) mode. Chromatography was performed at a flow rate of 300 nL/min over nano-columns (75 μm ID × 25 cm) packed with Reprosil-Pur C18-AQ (3 μm, Dr. Maisch GmbH). Water and 80% acetonitrile, both containing 0.1% formic acid, served as solvents A and B, respectively. The gradient consisted of 2% to 30% solvent B for 72 minutes, 30% to 60% B for 34 minutes, 60% to 90% B for 2 minutes, and 10 minutes of isocratic flow at 90% B. A full MS scan was acquired over 300 to 1400 m/z in the Orbitrap in in profile mode with a resolution of 70K, AGC target of 1×10^6^, and maximum injection time of 100 ms. The top 15 most intense ions (charge state 2+ through 6+) were selected for MS/MS by high-energy collision dissociation (HCD) at 30 NCE, and fragmentation spectra were acquired in the Orbitrap in centroid mode with a resolution of 17.5K, AGC target of 1×10^5^, isolation window of 2 m/z and maximum injection time of 50 ms. Dynamic exclusion of 40 s was used. Data was processed in Proteome Discoverer 2.2 (Thermo Fisher Scientific) using the Sequest-HT node to search MS/MS spectra against the UniProtKB/SwissProt (organism: *Mus musculus*) database along with a contaminant database. Trypsin was selected as the protease with a maximum of 2 missed cleavages. For the proteome data, the search parameters were as follows: 10 ppm precursor ion mass tolerance; 0.05 Da fragment ion mass tolerance; carbamidomethylation (+57.021 Da to Cys) as a static modification; and oxidation (+15.995 Da to Met) and acetylation (+42.011 Da to protein N-terminus) as variable modifications. The Percolator node was used with default parameters and data were filtered for less than 1% FDR at the peptide level.

### qRT-PCR.

Following fluorescent sorting of AT1 cells from mouse lungs, RNA isolation was performed with the PureLink RNA Micro kit (Thermo Fisher Scientific). The SuperScript IV First-Strand synthesis system (Invitrogen) was used to generate cDNA and real-time qPCR was performed with Power SYBR Green Master Mix (Thermo Fisher Scientific) on a QuantStudio 7 PCR System (Applied Biosystems).

### AT1 cell culture model, IHC, and morphology analysis.

Lineage-traced AT1 cells were obtained following FACS and were then placed into an organoid growth medium containing DMEM F12 (Thermo Fisher Scientific) and growth factors including bovine pituitary extract, cholera toxin, FBS, gentamicin, retinoic acid, insulin, transferrin (all from Lonza), and human epithelial growth factor (Peprotech), as previously described (58, 67). A total of 25,000 cells per well were plated onto 24-well plates that were coated with fibronectin (5 μg/cm^2^, Thermo Fisher Scientific) or Col4 (10 μg/cm^2^, Corning). To evaluate the effects of exogenous TGF-β ligand or inhibition of TGF-β signaling, TGF-β1 (7.5 ng/mL, Peprotech) or SB 431542 (10 μM, Abcam) were added at the time of plating. Cells were fixed in 2% PFA at days 2, 4, and 6 or collected in RNA lysis buffer. After fixation, cells were stained with anti-RAGE antibody (rat, R&D, MAB1179, 1:100) and the entire well was imaged with the EVOS FL Auto2 Imaging System at 20 × magnification. Only cells that were entirely within the image frame and were not adhered to an adjacent cell were assessed. Using Fiji software, cell area and roundness were quantified.

### Statistics.

All statistics were performed with GraphPad Prism8 or in R Studio. The number of replicates and statistical test used is described in the figure legend, including 1-way and 2-way ANOVAs with Tukey’s post-test correction and *t* tests with and without Welch’s correction. *P* values are denoted within the graph with significance determined by *P* < 0.05.

### Study approval.

All studies were approved by and performed in accordance with the University of Pennsylvania Institutional Use and Animal Care Committee.

### Data availability.

Sequencing data generated in this study has been deposited to GEO with accession number GSE230268. Proteomics data is available at MassIVE with accession number MSV000091803. Supporting data value for the manuscript may be found in the Supporting Data Values file.

## Author contributions

DAC contributed to designing research studies, conducting experiments, acquiring data, analyzing data, and writing the manuscript. IJP designed research studies, conducted experiments, acquired data, and analyzed data. SZ designed and conducted experiments. JJK acquired and analyzed data. FCD designed research studies and analyzed data. AB and MPM were involved in population RNA-Seq study design, data acquisition, data analysis, and figure generation. ML and BAG were involved in proteomics study design, data acquisition, and data analysis. EEM contributed to designing research studies and writing the manuscript. All authors contributed to manuscript review and editing.

## Supplementary Material

Supplemental data

Supporting data values

## Version 1 03/15/2024

Electronic publication
