# Supplementary material for: TGF-β controls alveolar type 1 epithelial cell plasticity and alveolar matrisome gene transcription in mice
Source: J Clin Invest. 2024 Mar 15;134(6):e172095. doi: 10.1172/JCI172095 (PMC10947970; doi:10.1172/JCI172095)
Supplement: Supplemental data [file jci-134-172095-s008.pdf]

SUPPLEMENTAL FIGURES

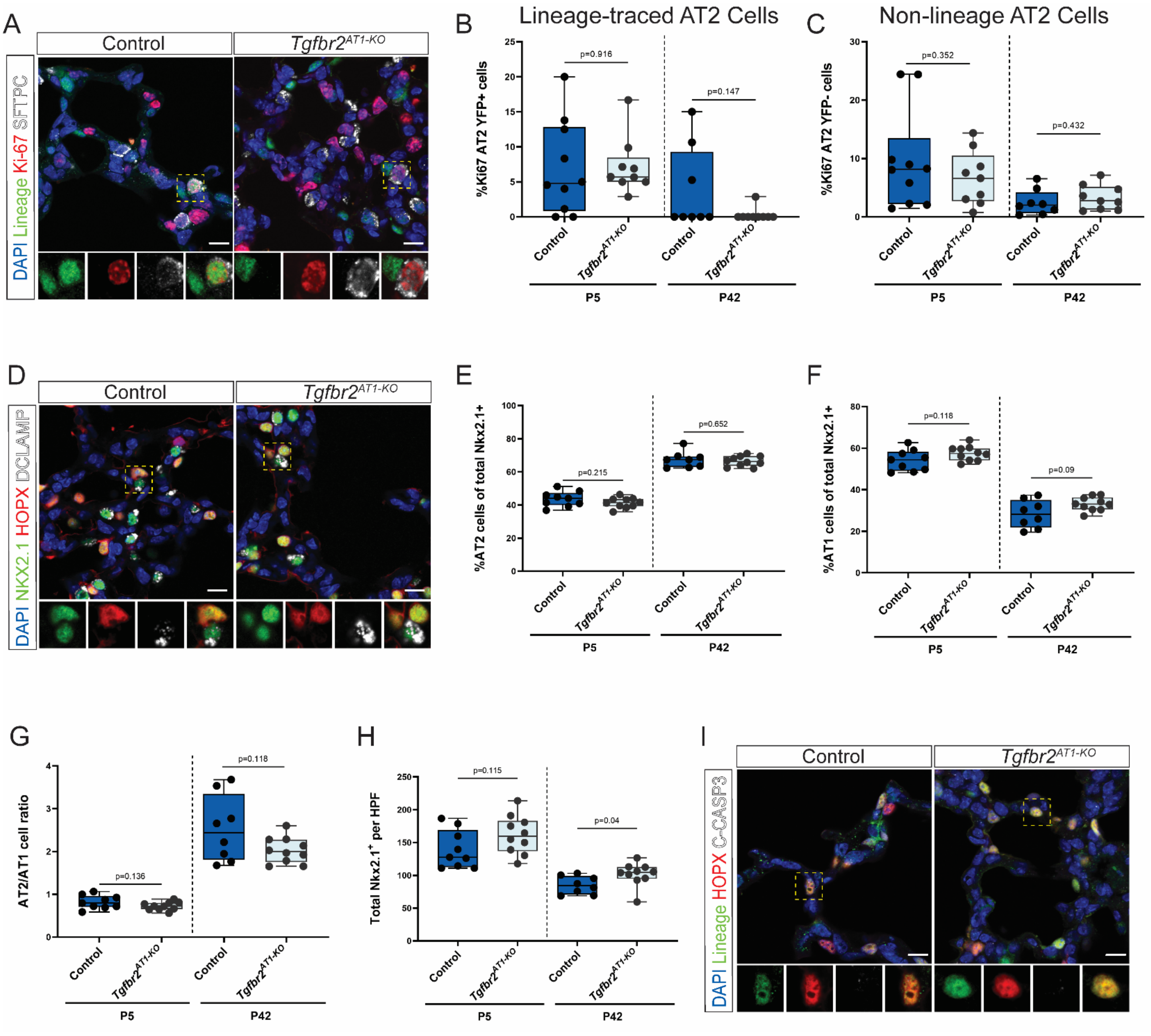

Figure S1. AT1 cell reprogramming in Tgfbr2$^{AT1\text{-}KO}$ mice does not affect AT2 cell proliferation, overall alveolar epithelial cell composition, or apoptosis.

A) IHC for EYFP, Ki67, and SFTPC indicate that there is no difference in lineage-traced AT2 cell proliferation across the groups. The yellow dashed box denotes the magnified region shown below the image and separated by fluorescence channel.

B) Quantification of AT2 cell proliferation from (A) denoting percent of cells that were EYFP+, SFTPC+, and Ki67+ at P5 (left) and P42 (right) or (C) percent cells that were EYFP-, SFTPC+, and Ki67+ by unpaired two-tailed t-tests with Welch's correction (n=8-10 per group).

D) IHC for NKX2.1, HOPX, and DCLAMP demonstrate no change in total AT2 or AT1 cell numbers after postnatal loss of *Tgfbr2*. The yellow dashed box denotes the magnified region shown below the image and separated by fluorescence channel.

E) Quantification of AT2 cell numbers from total NKX2.1+ cells in (D) denoting percent of cells that were NKX2.1+ and DCLAMP+ at P5 (left) and P42 (right) by unpaired two-tailed t-tests with Welch's correction (n=8-10 per group).

F) Quantification of AT1 cell numbers from total NKX2.1+ cells in (D) denoting percent of cells that were NKX2.1+ and HOPX+ at P5 (left) and P42 (right) by unpaired two-tailed t-tests with Welch's correction (n=8-10 per group).

G) Quantification of the AT2/AT1 cell ratio at P5 (left) and P42 (right) by unpaired two-tailed t-tests with Welch's correction (n=8-10 per group).

H) Quantification of total NKX2.1+ cells per high-powered field at P5 (left) and P42 (right) by unpaired two-tailed t-tests with Welch's correction (n=8-10 per group).

I) IHC for EYFP, HOPX, and cleaved caspase-3 do not indicate the presence of apoptotic cells after postnatal loss of *Tgfbr2*.

Each dot represents a single mouse. Scale bars denote 10 μm. P values are denoted above the plots.

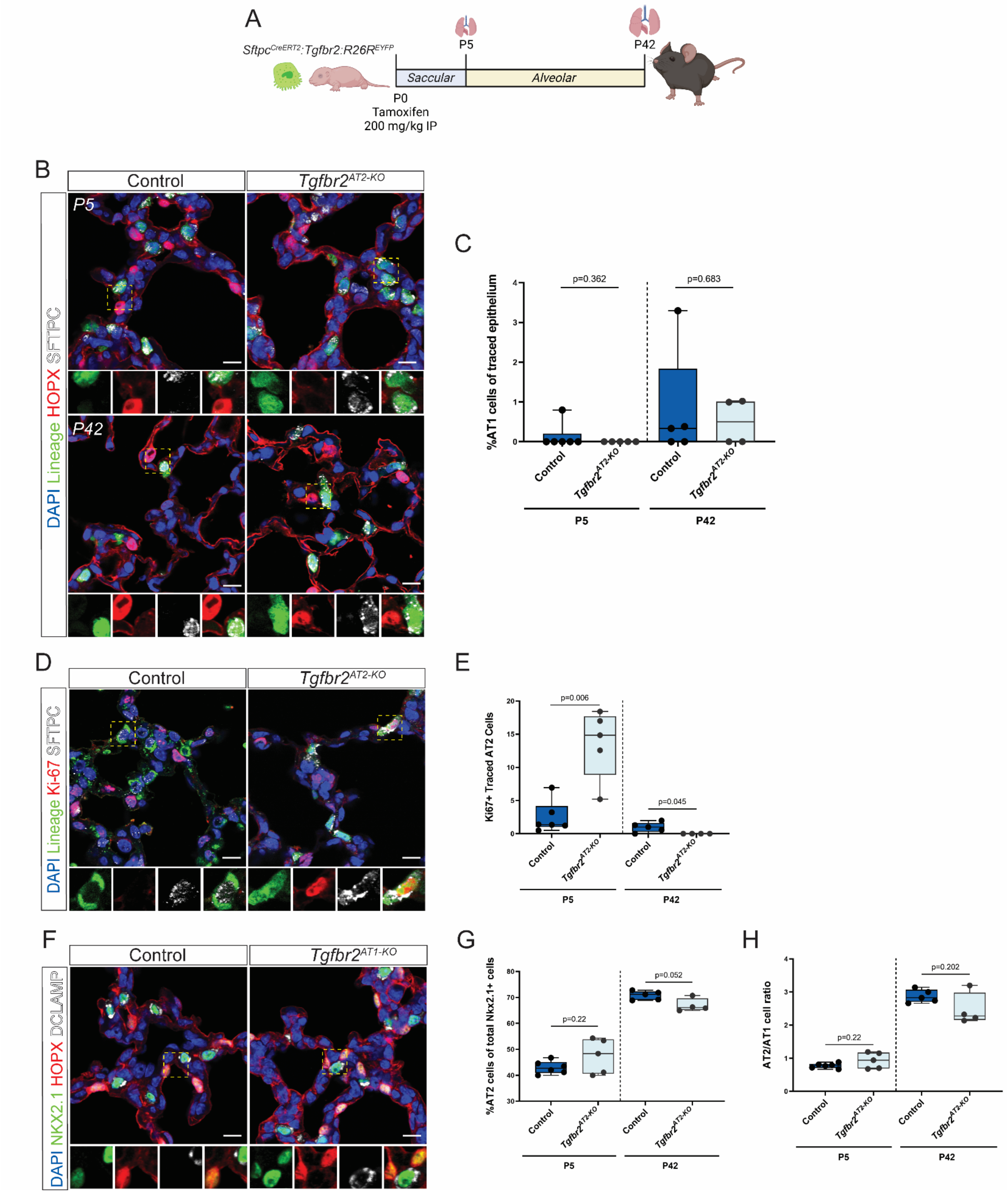

Figure S2. AT1 cells lacking Tgfbr2 do not reprogram during late lung development but do exhibit increased proliferation

A) In postnatal lineage-tracing experiments, control (*Sftpc*^{creERT2}*:R26R*^{EYFP}) and *Tgfbr2*$^{AT2\text{-}KO}$ newborn pups (P0) were injected with tamoxifen and the lungs were harvested at P5 and P42.

B) IHC for EYFP, HOPX, and SFTPC demonstrate little evidence of AT2:AT1 reprogramming through late lung development. The yellow dashed box denotes the magnified region shown below the image and separated by fluorescence channel.

C) Quantification of lineage tracing in (B) denoting percent of cells that were EYFP+ and HOPX+ at P5 (left) and P42 (right) by unpaired two-tailed t-tests with Welch's correction (n=4-6 per group).

D) IHC for EYFP, Ki67, and SFTPC indicate that AT2-KO pups at P5 demonstrate increased proliferation, which is dampened by P42. The yellow dashed box denotes the magnified region shown below the image and separated by fluorescence channel.

E) Quantification of AT2 cell proliferation from (D) denoting percent of cells that were EYFP+, SFTPC+, and Ki67+ at P5 (left) and P42 (right) by unpaired two-tailed t-tests with Welch's correction (n=4-6 per group).

F) IHC for NKX2.1, HOPX, and DCLAMP demonstrate no change in the epithelial cell composition after postnatal loss of *Tgfbr2* although there is a suggestion of a trend towards decreased %AT2 cells in AT2-KO animals at P42. The yellow dashed box denotes the magnified region shown below the image and separated by fluorescence channel.

G) Quantification of AT2 cell numbers from total NKX2.1+ cells in (F) denoting percent of cells that were NKX2.1+ and DCLAMP+ at P5 (left) and P42 (right) by unpaired two-tailed t-tests with Welch's correction (n=4-6 per group).

H) Quantification of the AT2/AT1 cell ratio at P5 (left) and P42 (right) by unpaired two-tailed t-tests with Welch's correction (n=4-6 per group).

Each dot represents a single mouse. Scale bars denote 10 µm. P values are denoted above the plots. Graphic in (A) created with BioRender.

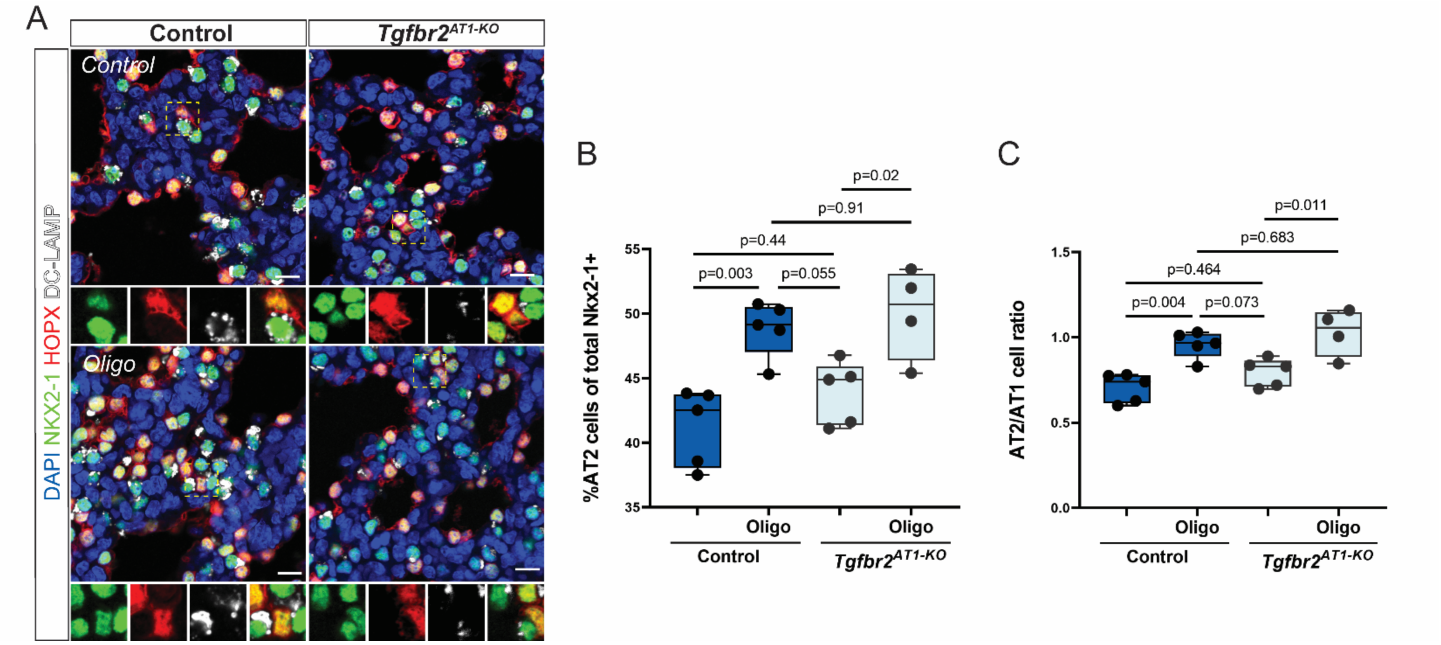

Figure S3. Prenatal oligohydramnios impacts alveolar epithelial cell composition

A) IHC for NKX2.1, HOPX, and DCLAMP in Control (left) or AT1-KO (right) animals subjected to oligohydramnios (bottom) or served as littermate controls (top) demonstrate that oligohydramnios results in increased AT2 cell composition which was not seen with loss of *Tgfbr2* in AT1 cells, alone. The yellow dashed box denotes the magnified region shown below the image and separated by fluorescence channel.

B) Quantification of AT2 cell numbers from total Nkx2.1+ cells in (A) denoting percent of cells that were NKX2.1+ and DCLAMP+ at E18.5 in the treatment conditions listed above by two-way ANOVA with Tukey's post-test for multiple comparisons (n=4-5 per group).

C) Quantification of the AT2/AT1 cell ratio in (A) at E18.5 in the treatment conditions listed above by two-way ANOVA with Tukey's post-test for multiple comparisons (n=4-5 per group).

Each dot represents a single mouse. Scale bars denote 10 μm. P values are denoted above the plots.

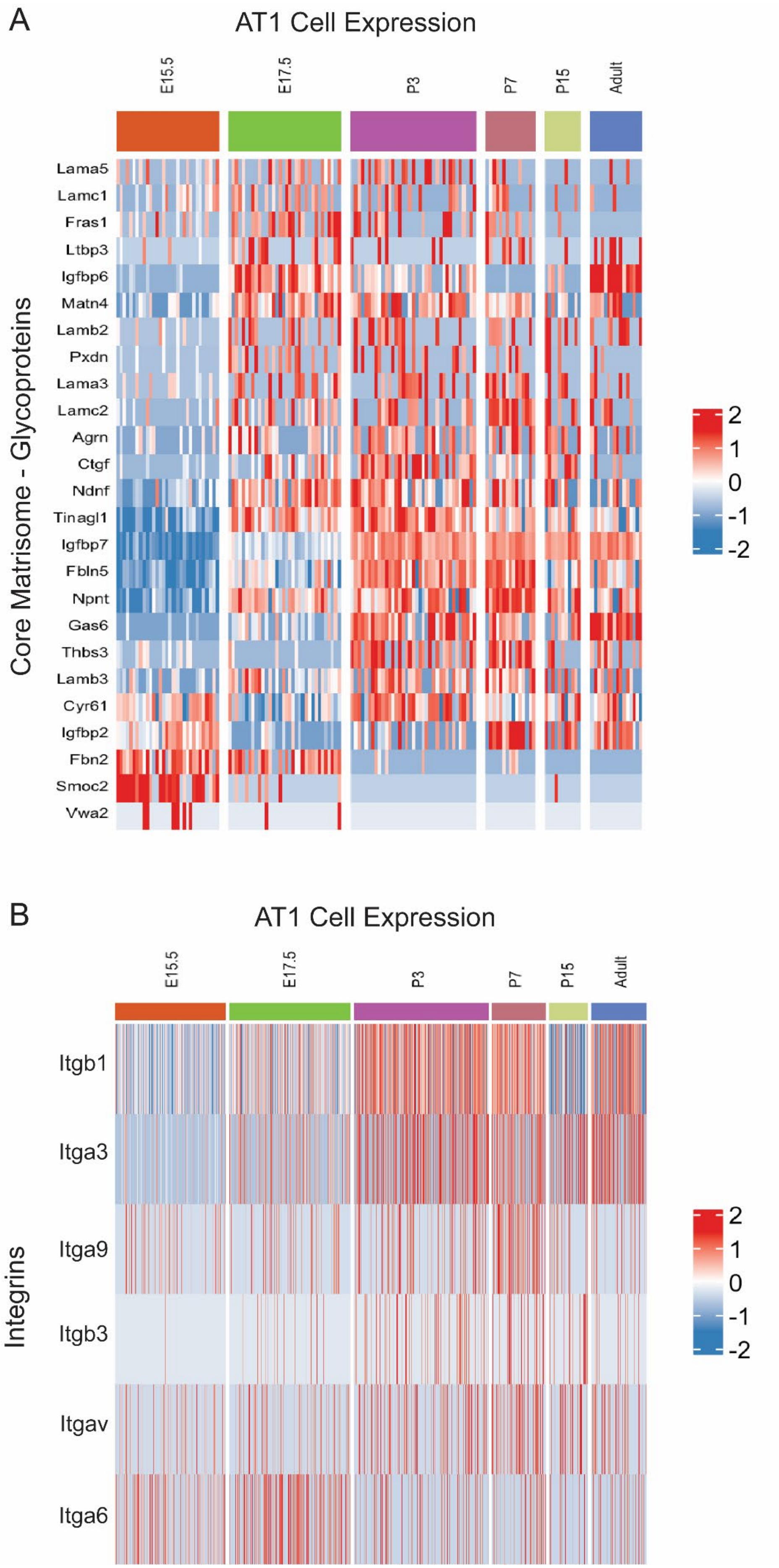
A
AT1 Cell Expression
E15.5
E17.5
P3
P7
P15
Adult
Core Matrisome - Glycoproteins
Lama5
Lamc1
Fras1
Ltbp3
Igfbp6
Matn4
Lamb2
Pxdn
Lama3
Lamc2
Agrn
Ctgf
Ndnf
Tinagl1
Igfbp7
Fbln5
Npnt
Gas6
Thbs3
Lamb3
Cyr61
Igfbp2
Fbn2
Smoc2
Vwa2
2
1
0
-1
-2
B
AT1 Cell Expression
E15.5
E17.5
P3
P7
P15
Adult
Integrins
Itgb1
Itga3
Itga9
Itgb3
Itgav
Itga6
2
1
0
-1
-2

Figure S4. AT1 cells exhibit a dynamic expression pattern for multiple matrisome and integrin genes starting in late lung development.

A) Evaluation of previously generated scRNA-seq data across late lung development indicates that AT1-enriched glycoprotein and (B) integrin genes become highly expressed in the postnatal period indicating a role for AT1 cells in the active production and remodeling of the pulmonary matrisome across the lifespan.

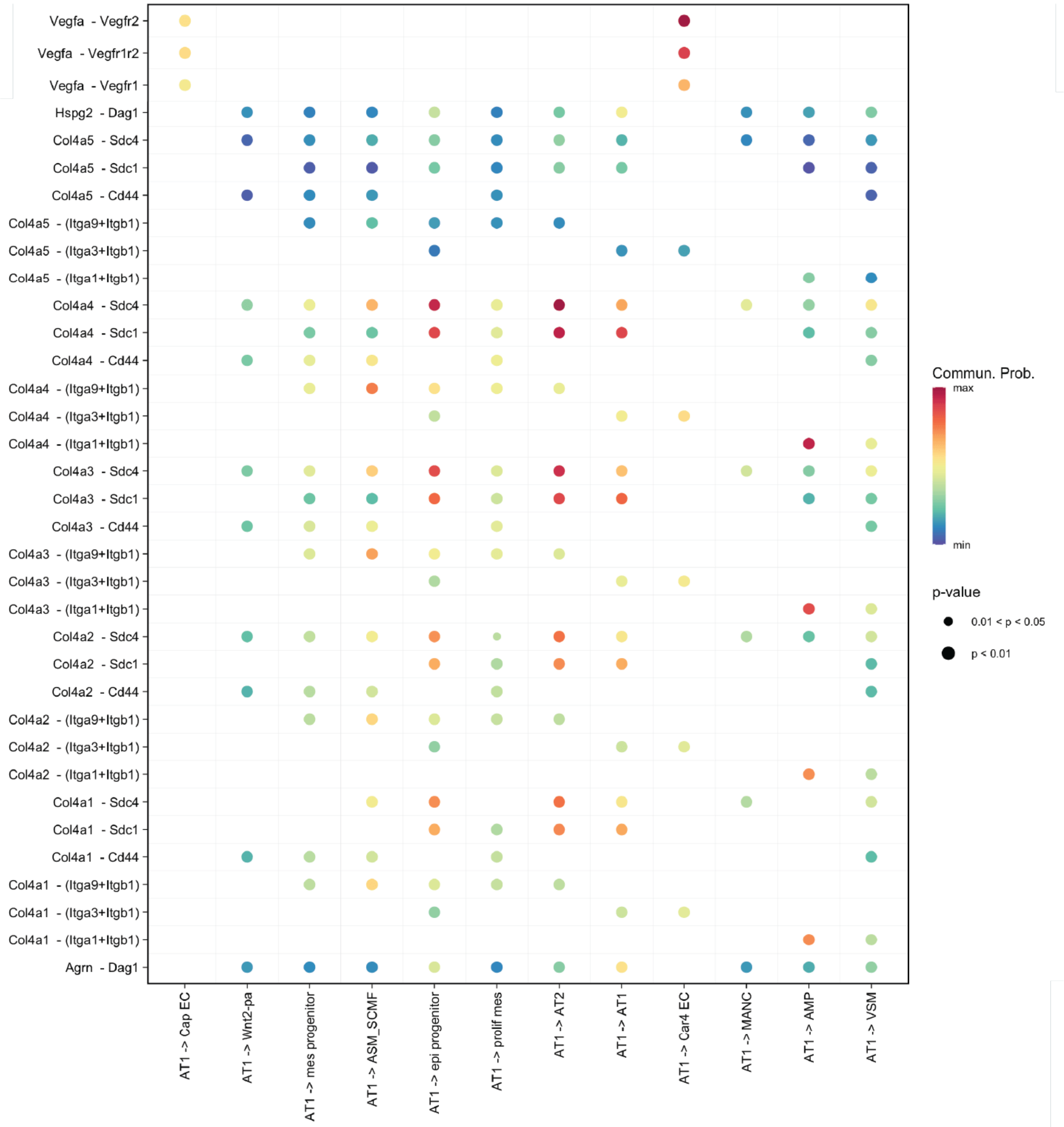

Figure S5. Receptor-ligand analysis at P3 reveals that AT1 cells are hubs for communication during development that rely on AT1-specific core matrisome expression

Cellchat receptor ligand-analysis of scRNA-seq data at P3 derived from Zepp et al., that identifies signals derived from AT1 cells including AT1-specific collagen IV subtypes (*col4a3, col4a4, col4a5*) and their binding to

integrins and other receptors of the neighboring epithelium and mesenchyme (Wnt2 progenitor cells – Wnt2-pa; mesenchymal progenitors - mes progenitor; proliferating mesenchyme – proli mes; mesenchymal alveolar niche cell – MANC; Axin2+ Myofibrogenic Progenitor – AMP; vascular smooth muscle - VSM. Also shown is Vegf signaling important for intercellular communication with endothelial cells (Capillary endothelial cells – Cap EC and Car4+ CAP2 endothelial cells – Car4 EC). Size of dot denotes p-value and communication probability denoted as a gradient from blue (minimal) to red (maximal).

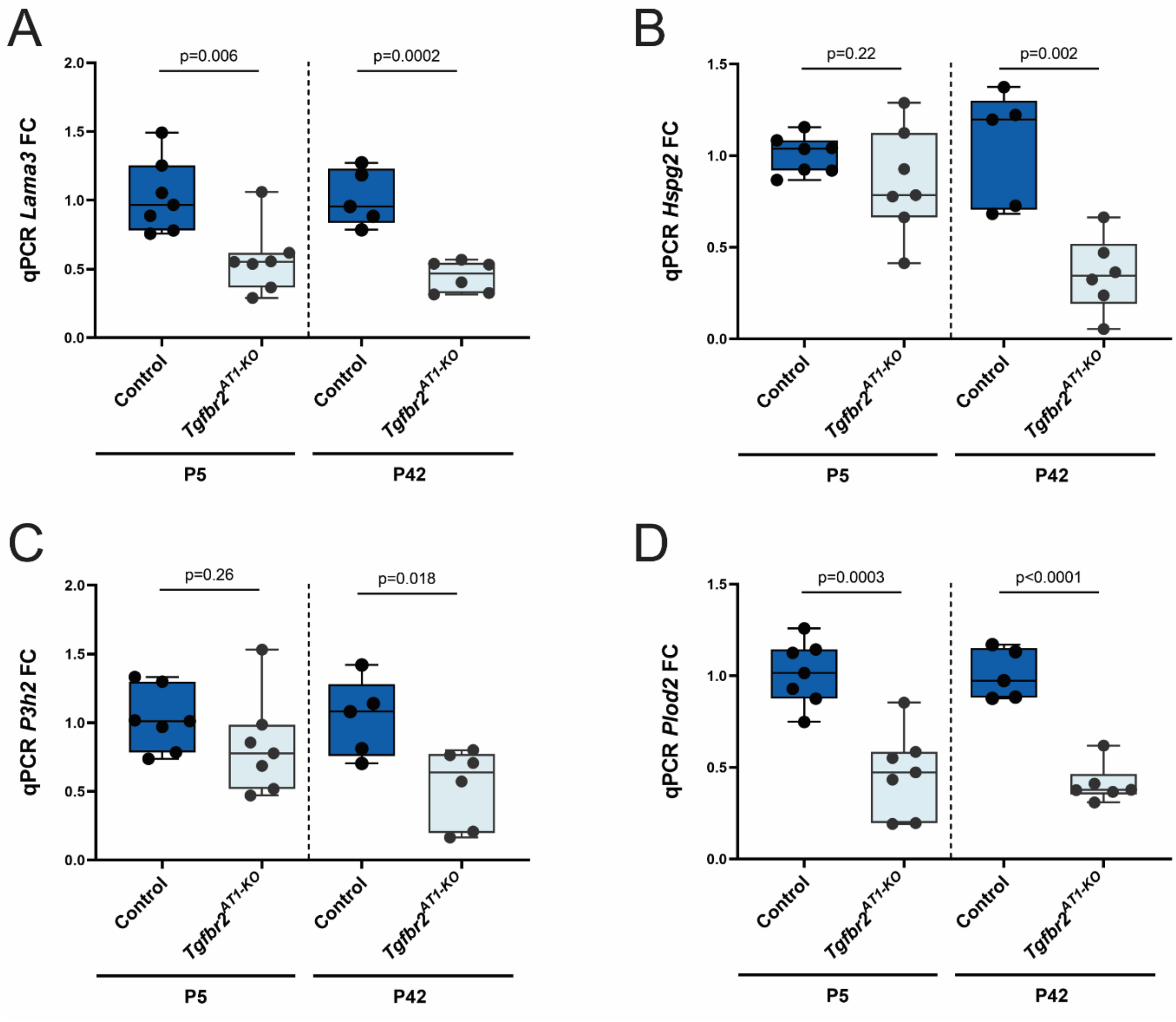

Figure S6. Tgfbr2^{AT1-KO} cells exhibit attenuated glycoprotein, proteoglycan, and matrisome regulatory enzyme expression

A) *Tgfbr2^{AT1-KO}* AT1 cells exhibit decreased RNA transcript expression for several AT1 cell-enriched core matrisome constituents including the glycoprotein (A) *Lama3,* the proteoglycan (B) *Hspg2*, and regulatory enzymes (C) *P3h2* and (D) *Plod2* at P5 that is more prominent at P42 (n=5-7, unpaired two-tailed t-test with Welch's correction).

Each dot represents a single mouse. P values are denoted above the plots.

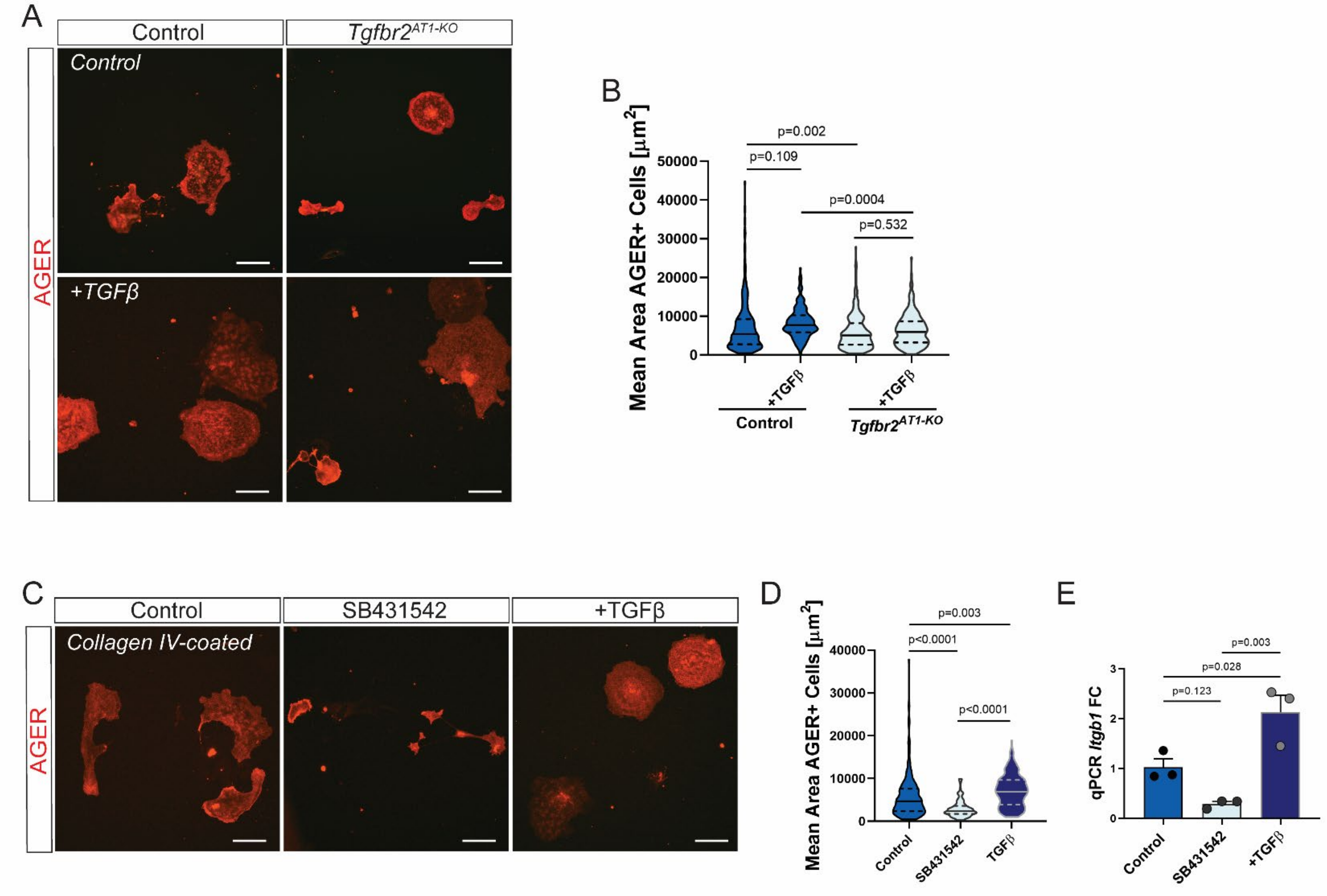

Figure S7. AT1-KO cells demonstrate decreased spreading capacity and TGFβ-mediated changes in cell spreading does not depend on plate substrate

A) ICC of RAGE+ (AGER+) cells from Control Hopx^{creERT2}:EYFP or AT1-KO pups treated with TGFβ ligand for 6 days. Cells from AT1-KO cells demonstrated decreased spreading capacity from control even in the presence of TGFβ ligand. Scale bars denote 100 μm.

B) Quantification of mean AGER+ cell area depicted in (A) by two-way ANOVA with Holms Sidak's test for multiple comparisons (n=230-384).

C) ICC of RAGE+ (AGER+) cells treated with TGFβ ligand or inhibitor in culture for 6 days on collagen IV-coated plates. Exogenous TGFβ resulted in increased cell spread whereas treatment with inhibitor led to decreased cell size. Scale bars denote 100 μm.

D) Quantification of mean AGER+ cell area depicted in (C) by one-way ANOVA with Holms Sidak's test for multiple comparisons (n=164-262).

E) Quantification of *Itgb1,* a collagen IV-binding integrin, transcript expression levels (fold change compared to GAPDH) indicating that TGFβ regulates its expression despite the change in cell culture substrate. (n=3 per group, one-way ANOVA with Tukey's multiple comparisons).
